# A brief glimpse of a tangled web in a small world: Tumor microenvironment

**DOI:** 10.3389/fmed.2022.1002715

**Published:** 2022-08-15

**Authors:** Iman M. Talaat, Byoungkwon Kim

**Affiliations:** ^1^Clinical Sciences Department, College of Medicine, University of Sharjah, Sharjah, United Arab Emirates; ^2^Department of Pathology, H.H. Sheikh Khalifa Specialty Hospital, Ras Al Khaimah, United Arab Emirates

**Keywords:** tumor microenvironment, Immune Checkpoint Inhibitors, immune system, network, immunotherapy

## Abstract

A tumor is a result of stepwise accumulation of genetic and epigenetic alterations. This notion has deepened the understanding of cancer biology and has introduced the era of targeted therapies. On the other hand, there have been a series of attempts of using the immune system to treat tumors, dating back to ancient history, to sporadic reports of inflamed tumors undergoing spontaneous regression. This was succeeded by modern immunotherapies and immune checkpoint inhibitors. The recent breakthrough has broadened the sight to other players within tumor tissue. Tumor microenvironment is a niche or a system orchestrating reciprocal and dynamic interaction of various types of cells including tumor cells and non-cellular components. The output of this complex communication dictates the functions of the constituent elements present within it. More complicated factors are biochemical and biophysical settings unique to TME. This mini review provides a brief guide on a range of factors to consider in the TME research.

## Introduction

The earliest form of cancer immunotherapy using infection started around 1550 BCE (1). In the modern era, an incidental observation of tumor regression after surgical wound infection was advanced into a more controlled approach using bacterial vaccines to treat sarcoma (2). This journey was then succeeded by application of Bacillus Calmette-Guerin (BCG), various types of oncolytic viruses and Immune Checkpoint Inhibitors (ICIs) (3). Substantial efficacy and superior safety profiles with tumor-agnostic features have immediately positioned ICIs in the main treatment arm in most advanced cancers. This has turned the focus from genetic and epigenetic alterations of tumor cells to immune cells. However, ICIs are no exception in primary or secondary resistance of drugs. This has led the investigators to place a heavier emphasis on other players and the surroundings of tumor cells. Long before the era of ICIs, histologic description of tumor tissues had already provided some insights in tumor surroundings. For instance, melanomas are characterized by fibrosis, melanophages (a type of macrophage), new blood vessels and infiltration of lymphocytes in and around the nests of dying tumor cells (4). Exuberant lymphoid reaction was the hallmark of colorectal cancer (CRC) with high microsatellite instability (MSI-high) (5). The study of CRC with MSI-high, either in Lynch syndrome or sporadic cases has indicated the hypermutator phenotype and MSI is still the most relevant predictive biomarker of ICIs currently (6). It is quite logical to speculate that the tumor mutational burden (TMB) follows MSI. However, the TMB is not a one-marker-fit-for-all (7). An example that displays this fact to the furthest extent was from an animal study where fibroblasts having inactivated TGF-β type II receptor induced precancerous lesions and carcinomas from an otherwise normal epithelium (8). With all these factors to consider, the center of attention always has been revolving around tumor cells. Environment is defined as the circumstances, objects, or conditions by which one is surrounded (9). The circumstances surrounding tumor cells theoretically ranges from ions, humoral factors and matrikines to various types of cells and tissues and even to host itself. Like the stem cell niche, tumor cells reside in their own niche or TME, and also have a reciprocal non-static spatiotemporal coordination with each other to regulate functions and differentiation of tumor cells and non-tumor cells, under the influence of specific physicochemical conditions (10–16). The current mini-review aims to cover as many attributes in this complex system, ranging from ions to cell and extracellular matrix (ECM), to physico-chemical properties of TME in an attempt to assist future studies.

## Definition of tumor microenvironment

The National Cancer Institute defines the TME as “The normal cells, molecules, and blood vessels that surround and feed a tumor cell. A tumor can change its microenvironment, and the microenvironment can affect how a tumor grows and spreads.” (17). This definition may appear simple at first, but encompasses the idea of reciprocal interaction and regulation of a tumor cell behavior. The most common ones are based on a structural view (18). Regularly emphasized is the dynamic nature of the cell population, such as the resident players and non-resident cellular components (19, 20). However, these definitions do not specifically identify other elements, such as tumor interstitial fluid, and physicochemical properties. To better depict a dynamic symbiotic system, “Seed and Soil,” an analogy of the stem cell niche, was introduced (14). “The TME comprises of a diverse cellular and acellular milieu, in which cancer stem cells (CSCs) develop and thrive, and various stromal and immune cells are recruited to form and maintain this self-sustained environment” (21). In that regard, the definition of “seed and soil” is comprehensive enough to cover all components in TME.

## Cellular component

Histologic observation of tumors shows cancer cells intricately mixed with various inflammatory cells, fibroblasts, fibrotic stroma and blood vessels. One of the most studied examples is colorectal cancer (CRC) with high microsatellite instability (MSI). The tumor cells exhibit morphologic alterations such as mucinous change, signet ring cell feature and medullary histology (22). The presence of other cellular players is observed such as high number of tumor infiltrating lymphocytes (TILs) and peritumoral lymphoid follicles reminiscent of the inflammatory pattern of Crohn's disease (5). There are many cases providing morphologic evidence of multiple players in tumor tissues (6). On the other hand, data-driven approach was able to characterize complex alterations from genes to transcription, and has brought in molecular classifications agnostic about morphology (23). However, immune cells are still the major focus in the era of ICIs, and the classification systems based on proportion of these cells have been proposed (24–26). Two tier system such as a hot tumor vs. a cold tumor is widely accepted one. A three tier system, such as immune infiltrated/inflamed, immune excluded, and immune silent/desert is also a commonly used method of classification (25).

Back to the role of each population in TME, cells are generally classified as tumor-promoting vs. tumor-suppressing (27) (Table 1). In this scheme, players are not simply dysfunctional in TME, but also actively suppress other immune cells and promote tumor cells, ranging from growth, invasion, metastasis to immune evasion (27). Members found to promote tumors are regulatory T cells (Tregs), myeloid-derived suppressor cells (MDSCs), M2 tumor-associated macrophages (TAMs), resident or derived from bone marrow/spleen, N2 tumor-associated neutrophils (TANs), cancer-associated fibroblasts (CAFs), tolerogenic dendritic cells (DCs) and more details are summarized in Table 1 (76–78). Once cells migrate into the TME, they are polarized or differentiated under the local condition, and in return, these cells accelerate the immune-suppressive and tumor-promoting environment (37). Hence, the state is not static but can be dynamic depending on the context or milieu of cytokines and signaling molecules. For example, M1 macrophage can turn into the M2 type and vice versa, while an intermediate form between M1 and M2 has been discovered (37). Proportion-wise, cancer-associated fibroblasts (CAFs) are the most abundant component in the tumor tissue (13). CAFs have a critical position in all steps, from tumor initiation to metastasis, and even being related to therapeutic resistance (8, 79). CAFs are derived from resident fibroblasts and other cells such as smooth muscle cells, vascular pericytes and bone marrow-derived mesenchymal cells, adipocytes and this process is caused by various factors [stromal cell-derived factor 1 (SDF1), platelet-derived growth factor (PDGF), transforming growth factor-β (TGF-β), fibroblast growth factor 2 (FGF2)] produced by tumor cells and immune cells (18, 80–83). CAFs then reciprocally promote tumor progression by production of growth factors (PDGF, TGF-β, epidermal growth factor (EGF), bone morphogenetic proteins (BMP) and C-X-C motif chemokine 12 (CXCL12), CXCL13) and these cells also stimulate angiogenesis by secreting vascular endothelial growth factor (VEGF), CXCL12 and FGF2 (72–75). Recently, focus was turned to rare cell populations in TME such as mast cells, basophils, eosinophils (84–86). The next-generation pathology, together with the single-cell analysis and systems pathology, will provide new insightful hints for developing effective therapeutic protocols targeting the TME (87, 88).

**Table 1:** Tumor-suppressing and tumor-promoting roles of diverse cells in tumor microenvironment.

	**Tumor-suppressing**	**Tumor-promoting**	**References**
T lymphocyte	• Th1→ ↑CTL, M1, NK • *via* IFN- γ, IL-2 • CTL??direct killing • CTL→ ↓angiogenesis *via* IFN-γ • Th9→ ↑CTL *via* IL-9 and ↑NK *via* IL-21 • Th17 recruit CTL, PMN, DC *via* CCL2, CCL7, CCL20, CXCL9, CXCL10	• Treg suppress CTL • Treg→ ↓costimulatory molecules on DC • Treg modulate homeostasis of NK *via* IL-2 • Treg→ ↑tumor growth *via* GFs • Treg→ ↑angiogenesis • Th2→ ↓Th1 and ↑M2 • Th17→ ↑angiogenesis	(25, 28–36)
B lymphocyte	• B cell as APC to T cell • B cell→ antibody & • IFN-γ→ ↑CTL	• Breg→ ↓CTL, macrophage, TAN *via* IL-10, TGF-β	(25)
Macrophage	• M1 cells as APC to Th1, NK • M1 produces inflammatory cytokine, ROS, RNS and ADCC→ killing tumor cells	• M2 produce IL-10→ induce PD-L1 on monocyte→ ↑infiltration of Treg and ↓CTL • M2→ ↑PD-1→ ↓macrophage phagocytosis via tumor PD-L1 • M2→ ↑PD-L2→ immune escape and tumor promotion *via* PD-1 • M2→ ↑tumor growth *via* EGF, FGF, PDGF, IL-4 • M2→ ↑angiogenesis *via* VEFG, IL-8, FGF, MMP-9	(25, 37–43)
Dendritic cell	• DC as APC and stimulate CTL *via* ICAM-1, CD86, CD40, CD80 • DC recruit naïve T cell *via* CCL17, CCL19, CCL22, IL-32 • DC stimulate Th1, CTL, NK *via* IL-12, IL-15 • DC→ ↑Ag expression by tumor *via* TNF-α, IL-6	• IL-10, TGF-β in TME→ ↑PD-1 on DC→ immune-suppressive DC • DC→ ↑Treg but ↓CTL, Th, macrophage, PMNs *via* IL-10, PDL1, IDO, Arginase-1	(44–50)
NKT cell	• NKT as APC *via* CD1d • NKT activates NK, DC, CTL *via* IL-12, CD40	• NKT II→ ↑M2, MDSC and ↓CTL via IL-4, IL-13	(51, 52)
NK cell	• NK kill tumor cells *via* ADCC, Fas-FasL, perforin-granzyme and cytokines (TNF, IFN-γ, GM-CSF, IL-6, and CCL5) • NK stimulate DCs *via* FLT3L	• TGF-β in TME→ ↑dysfunctional NK • NK→ ↑autonomous inhibitory checkpoint molecules (PD-1, TIGIT, CD96, TIM-3, LAG-3, CTLA-4, KIR2DL-1/2/3 and NKG2A)	(53–56)
Neutrophil	• N1 TANs kill tumor cells *via* ADCC and pro-inflammatory factors (IFN-γ, MMP-8) & ROS • N1 TAN recruit DC *via* CCL19, CCL20 and T cells *via* CXCL9, CXCL10 and stimulate CTL, NK *via* TNF-α	• Tumor cells produce GM-CSF→ PD-L1 expression in TAN *via* JAK/STAT pathway→ PD-L1+ TAN inhibit T-cell immunity (N2 TAN) • TAN suppress immune cells *via* Arginase-1, i-NOS • TAN recruit Treg *via* CCL17 • TAN→ ↑angiogenesis *via* MMP-9, VEGF	(25, 57–62)
Myeloid-Derived Suppressor Cell (MDSC)		• MDSC→ ↓immune cells *via* TGF-β, ROS, NO, Arginase-1, PGE-2 through PD-L1/PD-1 • MDSC→ ↓metabolites in TME • MDSC block lymphocyte homing *via* ↓e-selectin •MDSC→ ↑angiogenesis *via* VEGF	(56, 63–65)
Mast cell	• Mast cells regulate immune cells (T, B, APC) *via* cytokines	• Mast cells secrete angiogenic (VEGF-A, CXCL8, and MMP-9) and lymphangiogenic factors (VEGF-C and VEGF-F) • Mast cells secrete IL10→ ↑Treg in draining lymph nodes • Tumor cells secrete TNF-α→ ↑PD-L1 in mast cells *via* NF-κB pathway	(66–69)
Endothelial cell		• Tumor-derived HIF→ ↑endothelial cell sprouting *via* PDGF, EGF, VEGF, FGF, Ang2, IL-8→ ↑endothelial cell migration→ support nutrient and metabolite to tumor cells • ↓ICAM-1, VCAM on endotheleial cells→ ↓immune cell infiltration • ↑TGF-β, BMP in TME convert endothelial cells to CAF	(25, 70, 71)
Cancer Associated Fibroblast (CAF)		• Tumor cells secrete FGF, PDGF, SDF→ ↑CAF→ ↑PDGF, TGF-β→ ↑tumor growth • CAF→ immunosuppression *via* TGF-β • CAF→ ↑angiogenesis *via* VEGF, CXCL12 • CAF→ ↑MDSC recruitment *via* CCL7 • CAF→ glucosaminoglycans and MMP-2→ ↑tumor migration	(72–75)

*ADCC, antibody-dependent cellular cytotoxicity; Ag, antigen; Ang, angiopoietin; APC, antigen presenting cell; BMP, bone morphogenetic protein; Breg, B-regulatory lymphocyte; CAF, cancer-associated fibroblast; CAM, cell adhesion molecule; CAR, chimeric antigen receptor; CCL, CXCL, chemokines; CD, Cluster of differentiation; CTL, cytotoxic lymphocyte; DC, dendritic cell; ECM, extracellular matrix; EGF, epidermal growth factor; FasL, Fas-ligand; FGF, fibroblast growth factor; GF, growth factors; HIF-1, hypoxia-inducible factor-1; ICOS, inducible T-cell costimulator; IDO, Indoleamine 2, 3-dioxygenase; IL, interleukin; i-NOS, inducible nitric oxide synthase; M1, M1 macrophage; M2, M2 macrophage; MAB, monoclonal antibody; MDSC, myeloid-derived suppressor cell; MMP, matrix metalloproteinase; NK cell, natural killer cell; NKT cell, natural killer T cell; NKT II; type II NKT cells; NO, nitric oxide; PDL-1, programmed death-ligand-1; PGE2, prostaglandin E2; PMN, Polymorphonuclear neutrophil; RNS, reactive nitrogen species; ROS, reactive oxygen species; TAN, Tumor associated neutrophil; N2 TAN, N2 type tumor associated neutrophil; TGF-β, transforming growth factor-β; Th, T helper lymphocyte; Th1, type 1 T helper lymphocyte; Th17, T helper lymphocyte 17; Th2, type 1 T helper lymphocyte; Th9, T helper lymphocyte 9; TLR, Toll-like receptors; TME, tumor microenvironment; TNF-α, tumor necrosis factor-α; TRAIL, TNF-related apoptosis-inducing ligand; Treg, T regulatory lymphocyte; VEGF, vascular endothelial growth factor;→, influence; ↑, increase; ↓, decrease*.

## Extracellular matrix

Tumor stroma shows fibrosis or even desmoplasia in certain types of tumors, such as biliary cancer and gray-colored myxoid change, likely due to the ECM alteration (89, 90). ECM undergoes a remodeling process in physiologic and pathologic conditions, and it is an intricate phenomenon involving more than 700 proteins (91, 92). The characteristics of the remodeled ECM eventually affect the fate of cells (91, 92). The major alterations of tumor ECM are degradation, stiffening and physical remodeling (18, 93). In TME, acidic condition, excessive amount of proteases [i.e., matrix metalloproteases (MMPs), disintegrin and metalloproteinases (ADAMs), disintegrin and metalloproteinases with thrombospondin motifs (ADAMTS)] and production of reactive oxygen species (ROS) from tumor cells, CAF, TAN and TAM cause degradation of ECM (18). During this process, Extracellular Matrix-Derived Fragments are produced. These undertake active biological functions as matrikines leading to various effects such as acceleration of matrix production, promoting or suppressing tumor progression and angiogenesis (93, 94). Neoplastic tumors are stiffer than adjacent normal tissues and this is due to an excessive laydown of ECM and altered post-translational modification (PTM) (18). At first, CAFs secrete ECM in excess, including collagens, glycoproteins, proteoglycans, and polysaccharides (18). Then, the hypoxic condition enhances the cross-linking *via* production of lysyl oxidase (LOX) and transglutaminase from CAGs (95, 96). These modified rigid collagen fibrils are known to facilitate tumor cell migration and progression (97–100). In addition to the structural changes, PTM of ECM directly controls the tumor cell behavior by modulating the function of various growth factors embedded in the matrix (46, 101–103). For example, heparan sulfate proteoglycans (HSPGs) have different binding and releasing capacity of growth factors, depending on the sulphation pattern. This pattern is modified by the enzyme called endosulphatase (Sulf). In tumor tissue, the isotypes of Sulf are differentially expressed that the sulphation pattern made by Sulf1 inhibits the signaling pathways promoting tumors, while contrastingly, the other formed by Sulf2 enhances them (101, 102). Altered glycosylation patterns are reported in tumor tissues, and are currently under research (22, 104, 105). Lastly, mechanical force causes physical remodeling of the ECM, and makes fibers aligned to make routes for tumor cell migration (93). In TME, the ECM is continuously remodeled in terms of the amount, structure and chemical properties and this process shapes the interplay of the components modulating the fate of tumor cells in their progression (93). High-throughput proteomics approach is expected to acquire more insight from this process (91, 106).

## Biochemical component

One of the approaches to understand the biochemical property of TME is to look into the fluid of tumor or tumor interstitial fluid (TIF) (107, 108). TIF is characterized by high P_CO2_, low P_O2_ and low pH, and these parameters are linked with each other (11, 12). Hypoxia in tumor tissues is the major contributor to acidic environment. Rapid proliferation of tumor cells and insufficient oxygen supply cause hypoxia. This condition reprograms tumor cells favoring aerobic glycolysis with production of lactate (109). Major regulators in this process are hypoxia-inducible factor (HIF)-1α, c-Myc, and p53 (110–114). Hypoxia induces inhibition of prolyl-hydroxylases and this stabilizes the HIFs. HIF-1α switches metabolisms in tumor by upregulating the transcription of enzymes of glycolysis, such as hexokinase 1/2 (HK I/II) and pyruvate kinase isoenzyme M2 (PKM2), glucose transporters (Glut) such as Glut-1 and 3, alongside other genes inhibiting oxidative phosphorylation (115–118). As the dimer form of PKM2 prevails in the tumor, glucose metabolism is shifted to lactate production (118, 119). Abnormal vessels are unable to clear hydrogen ions effectively and hydration of CO_2_ by carbonic anhydrase IX in hypoxic areas further increase acidity (120). This altered biochemical environment reconditions the cells under its influence forming a selective pressure which favors cancer cells over normal cells (120–128). This situation promotes tumorigenesis, tumor progression and immune evasion and is related with a poor clinical prognosis and resistance to therapy. Recently reported findings suggest that the lactic acid not only intensifies acidity but also directly impacts cellular signaling pathways preferentially polarizing TAM to M2 type (129).

What about the ions in TME? Previous studies have shown that the concentration of ions in TIF is similar to that in plasma (130). Recently, this notion has been revisited. More sophisticated analysis revealed that the potassium concentration is higher in TIF, while other ions such as sodium, chloride and magnesium remain within normal range (131). Higher potassium level was found to suppress activation and effector function of T cells (131). A starvation response is induced by local hyperkalemia, and this in turn reduces nutrient uptake, resulting in the imbalance of Acetyl Co-A (AcCoA) level in subcellular compartments (132). In this setting, mitochondrial AcCoA is relatively higher than nucleocytosolic AcCoA, and this disproportionate state causes reduction of histone acetylation promoting stemness of T cells, eventually impeding the activation of effector genes (132).

ROS are known as the byproduct of hypoxic environment produced by tumor cells in TME, and the up-to-date interpretation is that ROS are not only radicals having damaging effect, but also have diverse biologic effects such as stabilization of HIFs to promote angiogenesis, activation of cell proliferation, as well as survival pathways, metabolic reprogramming, differentiation of CAFs and deregulation of immune cells (133). Reactive Nitrogen Species (RNS) are also rich in TME, due to an increase in arginine metabolism within tumor cells and tumor-infiltrating myeloid cells (134). RNS causes nitration of chemokine (C-C motif) ligand 2 (CCL2), and this modification suppresses infiltration and effector function of lymphocytes (134, 135).

Altered metabolic condition is a common survival strategy by tumor cells (136–139). Clinically, cachexia represents increased catabolic status to feed cancer cells (140, 141). Abnormally increased anabolism is also seen in cancer patients. Non-Islet Cell Tumor Hypoglycemia (NICTH) is a paraneoplastic syndrome where non-endocrine tumors cause hypoglycemia, while promoting anabolism of tumor cells by aberrantly producing insulin-like growth factor II (IGF-II), insulin receptor antibodies and various cytokines (tumor necrosis factor-α, interleukin-1 and−6) (142–145). Metabolic condition comes into play at microscopic level as well. As immune cells enter into tumor tissue, those cells face hypoglycemia and a scant amount of essential amino acids including glutamine and lipids. This condition hinders all steps of immune cell functions such as infiltration, proliferation and effector because these tasks have great demand for energy, nutrition and metabolic reprogramming (136–139). This competitive condition places the immune system in an anergy and exhaustion state (146, 147).

Extracellular vesicles (EVs) are rich in TIF (148). EVs such as exosomes, microvesicles, and apoptotic bodies carry active signaling and regulatory molecules like mRMA, miRNA, signaling proteins, microRNAs (miRNAs), long non-coding RNAs (LncRNAs), and circular RNAs (circRNAs) (149–151). All types of cells including cancer stem cells are known to secrete them (152, 153). Isolated EVs enriched in TME have the capability of promoting angiogenesis, modulating immune cells, enhancing tumor migration and epithelial-mesenchymal transition (EMT), metastasis and increasing drug resistance (148, 154, 155). However, EVs in TIF are not always tumor-promoting. Some EVs were found to exhibit anti-tumor effects (156, 157). This concept can be applied to patient treatment *via* an EV engineering. EVs derived from proven fighters such as active TILs and chimeric antigen receptor (CAR)-T cells may potentially recondition dysfunctional or anergic immune cells in tumor tissue (158–162). There are other humoral factors not mentioned here. Proteomic approach is expected to find unique signatures of TIF and further develop our understanding of the complex nature of TME.

## Biophysical component

Highly cellular tumors like lymphoma, seminoma, and Ewing sarcoma frequently present characteristic bulging cut surfaces. These features are related to an increased pressure inside tumor tissue (163). High tissue pressure is due to an increase in the proliferation and migration of tumor cells, alteration of ECM and increased interstitial fluid pressure (IFP) (163). The increased IFP is caused by the abnormal vessels having higher permeability, lack of pericytes, vascular compression by tumor growth and abundant ECM (164–167). IFP is elevated by 10–40 mmHg in tumor tissues (168, 169). Increased IFP generates an outward tissue flow and cell velocity flow, which hinders an inward penetration of cells, antibodies and drugs (164, 165, 170, 171). Interestingly, high pressure itself has been shown to enhance tumor proliferation and is often related to a poor clinical outcome (172–174). Vascular endothelial growth factor inhibitors, pegylated human recombinant hyaluronidase-α, collagenase and angiotensin inhibitors are suggested for potential drugs which can reduce IFP and promote the delivery of various molecules into tumor tissues (165). Migration and homing of immune cells is an entrenched process involving various chemokines, gradients and APC interaction (175–179). However, movement of immune cells under high IFP and altered ECM are not well studied, requiring further research.

## Conclusion

The main stream in cancer research has been about decoding genetic and epigenetic alterations in tumor cells. This scheme has been powerful to understand the nature of cancer diseases, and has led to the discovery of means to restore it. Meanwhile, a distinct course of ideas appeared long ago from the ancient time to the modern concept of immunotherapies and ICIs. This different perspective has widened sight to other attributes within tumor tissue. TME is a system consisting of a reciprocal communication network among components under unique physicochemical conditions. This process influences all components and the output influences TME in an iterative way. Various attempts such as data-driven approaches will rapidly improve understanding of surroundings of tumor cells and lead to several discoveries of predictive biomarkers and an eventual control of resistance. Another aspect not discussed in this mini review is about the host factors such as host genetic makeup. Certain single nucleotide polymorphisms (SNPs) in genes of the immune system were found to affect cancer susceptibility of an individual and these may also influence response to ICIs (180–182). There are case reports on renal cell carcinomas undergoing regression after transfusion of plasma from another patient of the same family (183, 184). This may indicate the presence of an inherited resistance to cancer. Even though these are still speculative and can be explained by other mechanisms, this macro-environment also needs to be considered in the dimension of future studies.

## Author contributions

BK drafted the initial version of the manuscript. IT reviewed it and added comments. Both authors contributed to the article and approved the submitted version.

## Conflict of interest

The authors declare that the research was conducted in the absence of any commercial or financial relationships that could be construed as a potential conflict of interest.

## Publisher's note

All claims expressed in this article are solely those of the authors and do not necessarily represent those of their affiliated organizations, or those of the publisher, the editors and the reviewers. Any product that may be evaluated in this article, or claim that may be made by its manufacturer, is not guaranteed or endorsed by the publisher.
